# A mouse-adapted CVA6 strain exhibits neurotropism and triggers systemic manifestations in a novel murine model

**DOI:** 10.1080/22221751.2022.2119166

**Published:** 2022-09-26

**Authors:** Dong Li, Tiantian Sun, Ling Tao, Wangquan Ji, Peiyu Zhu, Ruonan Liang, Yu Zhang, Shuaiyin Chen, Haiyan Yang, Yuefei Jin, Guangcai Duan

**Affiliations:** a Department of Epidemiology, College of Public Health, Zhengzhou University, Zhengzhou, People’s Republic of China; b Henan Key Laboratory of Molecular Medicine, Zhengzhou University, Zhengzhou, People’s Republic of China; c School of Public Health, Xinxiang Medical University, Xinxiang, People’s Republic of China

**Keywords:** Coxsackievirus A6, murine model, HFMD, oral infection, neurotropism

## Abstract

CVA6 is one of Enteroviruses causing worldwide epidemics of HFMD with neurological and systemic complications. A suitable animal model is necessary for studying the pathogenesis of CVA6 and evaluating antiviral and vaccine efficacy. In this study, we generated a mouse-adapted CVA6 strain that successfully infected 10-day-old ICR mice via oral route. All infected mice were paralyzed and died within 11 dpi. Analysis of pathological changes and virus loads in fourteen tissues showed that CVA6 triggered systematic damage similar to i.p. inoculation route. Unlike i.p. route, we detected oral and gastrointestinal lesions with the presence of viral antigens. Both specific anti-CVA6 serum and inactivated vaccines successfully generated immune protection in mice. Meanwhile, we also established a successful infection of CVA6 via i.p. and i.m. route in 10-day-old mice. After infection, mice developed remarkably neurological signs and systemic manifestations such as emaciation, polypnea, quadriplegia, depilation and even death. Through i.p. inoculation, pathological examination showed brain and spinal cord damage caused by the virus infection with neuronal reduction, apoptosis, astrocyte activation, and recruitment of neutrophils and monocytes. Following neurological manifestation, the CVA6 infection became systemic, and high viral loads were detected in multiple organs along with morphological changes and inflammation. Moreover, analysis of spleen cells by FACS indicated that CVA6 led to immune system activation, which further contributed to systemic inflammation. Taken together, our novel murine model of CVA6 provides a useful tool for studying the pathogenesis and evaluating antiviral and vaccine efficacy.

## Introduction

Hand, foot, and mouth disease (HFMD) is a highly contagious disease affecting infants and children worldwide, especially in Asia Pacific region [1]. Although Enterovirus A71 (EVA71) and Coxsackievirus (CV) A16 were considered two major pathogens of HFMD in the past [2,3]. With the use of EVA71 vaccine, the pathogen spectrum of HFMD has been changed in recent years. Nowadays, CVA6 is one of the significant agents to cause HFMD outbreaks globally. CV-A6 (71.1%) has become the predominant EV serotype in the epidemic of HFMD in Shanghai since 2017 [4]. In the United Kingdom, the proportion of samples typed as CVA6 increased sharply, from ≈1% in 2007–2008 to 10% in 2016–2017, and the proportion of samples typed as EVA71 decreased [5]. CVA6 was also frequently detected in Europe [6–8], Asia [9–11], and North America [12,13], and CVA6 has replaced EVA71 and CVA16 as the primary pathogen of some HFMD outbreaks [14]. Importantly, CVA6 was also found to be associated with an increased incidence of HFMD in adults [15]. Therefore, CVA6 infection has become a serious public health issue worldwide. The main clinical manifestations of CVA6 infection are herpangina and skin rash [16]. Recent studies reported that CVA6 infections developed serious complications, such as aseptic meningitis, brainstem encephalitis (BE), acute flaccid paralysis [17–19], and even fatal pulmonary edema [3]. As yet, there is no specific drug or vaccine to enable the treatment or prevention of clinical CVA6 infection, and the body of knowledge on CVA6 infection, pathogenesis, and immunogenicity are limited. Establishing animal models with typical clinical symptoms is one of the crucial steps in efficacy and safety evaluation of antiviral drugs and vaccines.

Overall, Enterovirus animal models mainly consist of the models of mouse-adaptation models [20], murine [21], gerbil [22], immunodeficient mice [23], non-human primate [24], tree shrew [25] and transgenic mice models [26]. SCARB2, a functional receptor for EVA71 entry, has an essential effect on EVA71 infection, indicated by our recent research on Enterovirus infection [26]. For most of the above models, the main routes of infection are through intraperitoneal (i.p.), intramuscular (i.m.), and intracranial (i.c.) injection. Indeed, establishing the orally infected model for Enterovirus infection is extremely difficult. Previous studies reported that one-day-old neonatal BALB/c mice and five-day-old ICR were susceptible to infection with non-mouse-adapted CVA6 via i.p., i.m. and i.c. inoculation. However, the neonatal mice are too fragile and therefore are not suitable to be used in animal experiments [27,28]. Another study introduced a 10-day-old murine model of CVA6 with mouse-adapted strains via i.p. inoculation [20]. Nevertheless, because i.p., i.m. or i.c. injection is not the natural route for Enterovirus infection, the application of these models is extremely limited and does not fully reveal the pathogenesis of oral infection pathways.

In this study, the skeletal muscles of the younger neonatal mice with CVA6 infection were collected to attack the elder newborn mice gradually, and then were utilized to prepare a mouse-adapted strain capable of stably causing severe illness in 10-day-old mice via oral infection. Therefore, the present study establishes a murine model to develop CVA6 therapeutic drugs and inactivated vaccines and provides a reference for researches on other types of Enterovirus models for elder newborn mice. We also studied systemic manifestations of CVA6-infected mice, indicating immunopathology and multi-organ damage were involved in the pathogenic process of CVA6.

## Materials and methods

### Ethics statements

Inbred, specific-pathogen-free (SPF) ICR mice were used to develop an animal model. All animal experiments were carried out strictly in accordance with the protocols approved by the Life Science Ethics Review Committee of Zhengzhou University (permission no: ZZUIRB2020-29).

### Cells and CVA6 strains

African green monkey kidney cell line (Vero 76, ATCC CRL-1587) (Shanghai, China) and human rhabdomyosarcoma cell line (RD) (the Chinese Academy of Sciences, Shanghai, China) were maintained in Dulbecco’s modified Eagle’s medium (DMEM) (Thermo Fisher Scientific Co., Ltd, Waltham, USA) supplemented with 10% fetal bovine serum (FBS) (Cegrogen Co., Ltd, Germany) at 37°C in a 5% CO_2_ humidified incubator. A CVA6 strain (GenBank Accession number: OM179765) was isolated from stool sample of an HFMD case. The 3-day-old mice were i.p. inoculated with CVA6, and their physiological states were observed every day. Once limb paralysis was occurred, the hind limb muscles were collected, and then were grinded by saline with liquid nitrogen for 3× times, and centrifuged at 12,000 rpm for 20 min. The supernatant was separated and filtered via a 0.22 µm filter membrane, After that, we i.p. inoculated the supernatant into the 5-day-old mice. Likewise, once limb paralysis was occurred, the hind limb muscles were collected, and then were grinded by saline with liquid nitrogen for 3× times, and centrifuged at 12,000 rpm for 20 min. The supernatant was separated and filtered via a 0.22 µm filter. Next, we i.p. inoculated the supernatant into 7-, 10-, 15-day-old mice. Finally, a mouse-adapted CVA6 strain capable of causing clinical symptoms above grade 4 in 10-day-old mice was generated. For the preparation of CVA6 working stocks, the 7-day-old mice were i.p. inoculated with the mouse-adapted CVA6 strain. Once limb paralysis was occurred, the hind limb muscles were collected, and then were grinded by saline with liquid nitrogen for 3× times, and centrifuged at 12,000 rpm for 20 min. The supernatant was separated and filtered via a 0.22 µm filter. CVA6 working stocks were stored at −80°C. The titre was quantified by the Reed-Muench method [29] to be 10^5^TCID_50_/mL.

### Full-length CVA6 sequencing and phylogenetic analysis

In order to obtain the whole genomic sequence of the CVA6 virus, virus RNA was extracted using Omega, MicroElute viral RNA kit. 750 ng of total RNA were reverse transcribed into cDNA with Hifair® II 1st Strand cDNA Synthesis Kit (Yeasen Co., Ltd, Shanghai, China). The whole length was divided into 12 segments and amplified respectively, and finally these segments were spliced together. The amplification primers used are shown in Table 1. Furthermore, the Molecular Evolutionary Genetic Analysis (MEGA) version 7.0 was used to perform the phylogenetic analysis of 29 CVA6 strains (9 strains from the GenBank database and 1 strain isolated in this paper) and other Enteroviruses strains based on the complete VP1 sequences.

**Table 1.** Primers used in this study for sequencing.

Forward primer	Reverse primer	Product length
TCCCAAGCTTATATGGTCAAGAAC	TTGACCCCTTGCTCATCCAC	2390
TGTCTTGGATGCTGGTGTCC	CTGCTATCTGGCTTAGGGGC	1473
AGACCTTTTGTGGCTGGATGA	GGGCAATTATACCGGTTGCC	764
CTCGCTCACTTTTGCCGTAA	TGGGCTACAGCGTTTGAAGT	603
TGCTCAAGAAACCCATCCTCC	AGCCAATTGCGTCTCAGGTT	1963
TACGGAATCTTTGTGCGCCT	GAGTTGGTTCAAAACCGGGC	1756
AATCCCCTTCTGTTGAGGCG	AGGGATCACAACCAACCCAA	662
TCAATAGACTGCTAGCGCGG	TCGGTCACTATAACCGCACG	701
TTAAAACAGCCTGTGGGTTGCA	ATCTACACTGGGGGAGTGCT	321
GTTGCAGTGGTCTCACTGGT	ACCCATTGGATCTCTCCTTGC	686
AGCAAGAACAGGCAAGGAGTA	ACCAGATTCCTGGTGGGGTT	394
CCATGACCCCCGCAGATAAG	ACCCCCACCAGTCATATTCAC	387

### Animal infection experiments

We developed an animal model of CVA6 infection based on dose, age and inoculation routes. Ten-day-old ICR mice were employed in dose-dependent experiments via i.p. inoculation with CVA6 (10^0^∼10^5^TCID_50_ per animal). For age-dependent experiments, mice were selected at 7, 10, and 15 days of age and i.p. inoculated with CVA6 (10^4^ TCID_50_). For the inoculation route experiments, 10-day old mice were inoculated via i.c., i.p., and i.m. route with CVA6 (10^4^ TCID_50_ per animal). To develop an oral infection model, 10-day-old suckling mice were inoculated by intragastric (i.g.) route with 10^5^ TCID_50_ CVA6. The mock-infected mice were inoculated with an equal volume of muscle homogenate supernatant from normal mice and kept in a separate cage from the infected mice. Each group included 10∼15 animals. The body weights, clinical signs, and survival rates of mock – or CVA6-infected mice were recorded for 15 dpi (days post-infection). The grade of clinical disease was scored as follows: 0, healthy; 1, lethargy and inactivity; 2, ataxic; 3, lose weight; 4, hind limb paralysis; 5, dying or death. The control mice were healthy during the experiments.

### Histopathological, immunohistochemical (IHC) analysis

The 10-day-old ICR mice were i.p. or i.g. inoculated with 10^4^ TCID_50_ CVA6 strains. At 5 dpi and 7 dpi, mock and infected mice were euthanized. The brains, spinal cords, lungs, limb muscles, hearts, kidneys, intestines, livers, stomachs, spleens, skins, upper jaws, down jaws, tongues, and claws samples were obtained and fixed in 10% paraformaldehyde for 48 h. After fixation, paraffin-embedded organs and tissues were cut into 5 μm sections and stained with hematoxylin and eosin (H&E). The expression of CVA6 VP1 (GeneTex Inc., Irvine, CA, USA) (Catalog number: GTX132346) in those tissues of mock- and CVA6-infected mice was detected by immunohistochemical (IHC) staining in accordance with a standard immunoperoxidase procedure as described previously [20]. Because the mouse-adapted CVA6 strain was generated from muscle tissue of mice, muscle slices were used as positive controls in this study. Tissue slices without incubating primary antibodies were regarded as negative controls (Figure S2). As mentioned above, paraffin-embedded brains and spinal cords were cut into 5 μm sections and Nissl’s staining was performed to detect the surviving neurons. H&E staining, IHC staining and Nissl’s staining technical service were provided by Service Biotech Co., Ltd. as described previously [30].

### Cytokines

Ten-day-old ICR mice were i.p. and i.g. inoculated with a lethal dose of CVA6, and brains, skeletal muscles, and serum were collected at 3, 5, and 7 dpi. Brains and skeletal muscles were quickly lyzed in a cold Radio-Immunoprecipitation Assay (RIPA) lysis buffer that was added with protease inhibitor cocktail (Beyotime Co., Ltd, Shanghai, China). The concentrations of total proteins were detected using the BCA protein assay kit (Biomed Co., Ltd, Beijing, China) according to the manufacturer’s instructions. The concentrations of IL-6, IL-10, TNF-α, IL-1β and MCP-1 in brains, skeletal muscles, and serum were determined with enzyme-linked immunosorbent assay (ELISA) detection kits (Biolegend Inc., CA, USA) according to the manufacturer’s instructions. The results were standardized with the concentrations of total proteins in each tissue.

### Quantitative real-time PCR

The copy numbers of CVA6 working stocks were determined by qRT-PCR using *in vitro*-transcribed RNA standards. RNA standards (10^1^–10^8^ copies) were transcribed from the CV-A6 VP1 gene cloned into a plasmid pET-28a (+) easy vector. For determination of viral load, total RNA was extracted from organs and tissues of control and infected mice using TRIzol reagent (Invitrogen Corporation, California, USA). 1 µg of total RNA was reverse transcribed into cDNA with Hifair® II 1st Strand cDNA Synthesis Kit (Yeasen, Co., Ltd, Shanghai, China). The forward primer VP1 F (5’-CAAGCTGCAGAAACGGGAG), reverse primer: (5’-GCTCCACACTCGCCTCATT) was performed with Hieff® qPCR SYBR Green Master Mix (Yeasen, Co., Ltd, Shanghai, China).

### Western blotting analysis

Briefly, total proteins from Vero cells and brains were extracted. Samples were separated by SDS-PAGE (Jingcai, Xian, China) and transferred into PVDF membranes (Immobilon-PSQ, Millipore, USA). Then membranes were blocked with 5% BD Difco^TM^ skim milk (BD Inc., New Jersey, USA) and incubated with primary antibodies overnight at 4°C. PVDF membranes were washed 3× times with PBST (PBS+0.1%Tween-20) and incubated with corresponding secondary antibodies for 1 h at room temperature. Afterward, the membranes were finally washed 3× times and blots were developed with ECL chemiluminescent substrate (Absin Bioscience, Inc., Shanghai, China). The following primary antibodies were used for Western blotting: anti-CVA6 VP1 antibody (GeneTex Inc., Irvine, CA, USA), anti-Caspase 3 and cleaved-Caspase 3 (CST Inc., Boston, USA), anti-β-actin (Proteintech, Inc, Chicago, USA), anti-GFAP (Absin Bioscience, Inc. Shanghai, China).

### Flow cytometry analysis

The 10-day-old ICR mice were i.g. inoculated with 10^5^ TCID_50_ CVA6 strains. At 1 dpi, 3 dpi, 5 dpi, and 7 dpi, mock- and CVA6-infected mice (*n* = 7–10) were euthanized. The brain and spleen cells were extracted and staining as described previously [31,32]. The antibodies used in FACS analysis were purchased from Biolegend Inc., CA, USA: APC-conjugated CD3 antibody (#100312), AF700-conjugated B220 antibody (#103210), BV711-conjugated CD4 antibody (#100549), Biotin anti-CD8 antibody (#100704), FITC-conjugated CD44 antibody (#103006), PE-conjugated CD69 antibody (#104507), FITC-conjugated CD25 antibody (#101907), FITC-conjugated Ly-6c antibody (#128006), PE-conjugated CD62L antibody (#104407), PE-conjugated CD11c antibody (#117308), PerCP/Cy5.5-conjugated Ly-6G antibody (#127615), APC-conjugated F4/80 antibody (#123115), APC/Cy7-conjugated CD45 antibody (#103116), PerCP/Cy5.5-conjugated IgM antibody (#406512), PE/Cyanine7 Streptavidin (#405206), anti-CD16/32 antibody (#101330). LIVE/DEAD Fixable Violet Dead Cell Stain Kit (Thermo Fisher Scientific Co., Ltd, Waltham, USA) was used to distinguish dead cells from living ones. Flow cytometry measurement was performed using the BD LSRFortessa FACS Canto and the data was analyzed by the FlowJoX software.

### Protective efficacy of antiserum and ribavirin

To study the protective effects of passive immunization, 10-day-old mice were i.g. inoculated with 10^2^ TCID_50_ CVA6 that was not enough to kill all the mice and their status was observed daily. The mice that were recovered from severity were sacrificed humanely, and blood samples were taken to obtain antiserum. We administered CVA6 antiserum with 2-fold serial dilution (2 to 256 folds) via i.p. injection into 10-day-old mice, while mock mice were administrated with an equal volume of normal serum (NS). Next day, a lethal dose of CVA6 was inoculated into the mice in both groups via i.g. route. The clinical signs and survival rates were monitored and recorded daily until 15 dpi. To evaluate the treatment with ribavirin *in vivo*, 10-day-old ICR mice were i.g. challenged with a lethal dose of CVA6. Within 1 h of inoculation, each mouse was i.p. injected with ribavirin (200 μg/mouse) or PBS, and the mortality rates and clinical symptoms were then monitored and recorded daily after infection until 15 dpi.

### Effects of active immunization on neonatal mice

To evaluate the immune-protective effects of active immunization, CVA6 viruses were purified using an ultrafiltration centrifuge tube (Amicon Ultra-15) and then inactivated at 56°C for 30 min, the CVA6-whole-virus inactivated candidate vaccine was prepared by adding the same amount of Imject^TM^ alum adjuvant. Newborn mice were divided into two groups. One group was inoculated with inactivated CVA6 whole-virus vaccine 50 μL on day 3 and day 7, and another group was immunized with an equal volume of muscle homogenate supernatant from normal mice. A lethal dose of CVA6 was i.g. injected into the 10-day-old immunized mice. The clinical illnesses of mice were recorded daily until 15 dpi.

### Statistical analysis

Data were presented as mean ± standard deviation (SD). Statistical analysis was performed with GraphPad Prism version 8.3 (GraphPad Software Inc, San Diego, CA, USA). The Mantel–Cox log rank test was used to compare the survival of different groups of mice. Differences in the body weights, and clinical scores were analyzed with two-way ANOVA. Differences in cytokines, viral loads, the number of neurons, and biochemical detection indicators of liver and kidney were determined using one-way ANOVA and unpaired student’s *t*-test. Adobe Illustrator CS6 software (Adobe, San Jose, CA, USA) was used for drawing. A *P* value less than 0.05 was considered as statistically significant in this study.

## Results

### Identification of CVA6 strain in clinical isolates from HFMD patients

**Figure 1.**
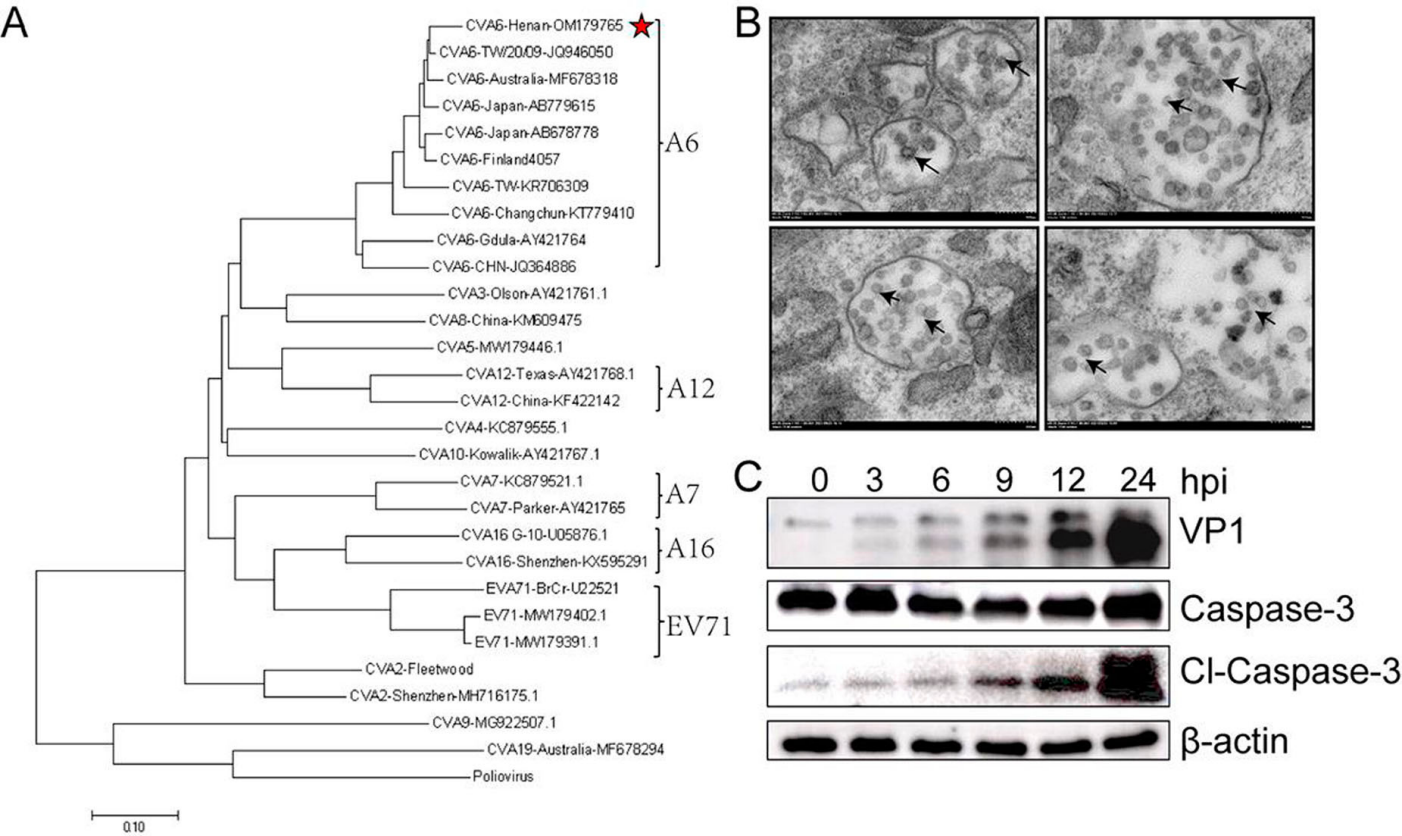
Identification of CVA6 strains in clinical isolates from HFMD patients. (A) A genetic evolutionary tree of CVA6 VP1 (926 bp) by using the neighbor-joining method. (B) Transmission electron micrograph of the infected Vero cells and CVA6 virions. The black arrows indicate spherical particles of the CVA6 virions with a diameter of 26–35 nm; (C) The relative expression of CVA6 VP1 and cleaved-Caspase-3.

RD cells were used to isolate viruses from stool samples of HFMD patients. After sequencing, a phylogenetic tree based on the capsid protein VP1 of Enteroviruses strains identified worldwide showed A6 type of this clinical isolates (Figure 1(A)). Through transmission electron microscope, we observed viral particles located in exosomes with a diameter of 26–35 nm (Figure 1(B)). In order to detect the virulence of CVA6 strain *in vitro*, the expression levels of VP1 and cl-Caspase-3 in Vero cells were measured. As shown in Figure 1(C), the expression levels of VP1 and cl-Caspase-3 exhibited a marked dose-dependent manner at the indicated doses.

### Establishment of the CVA6 infection mouse model

**Figure 2.**
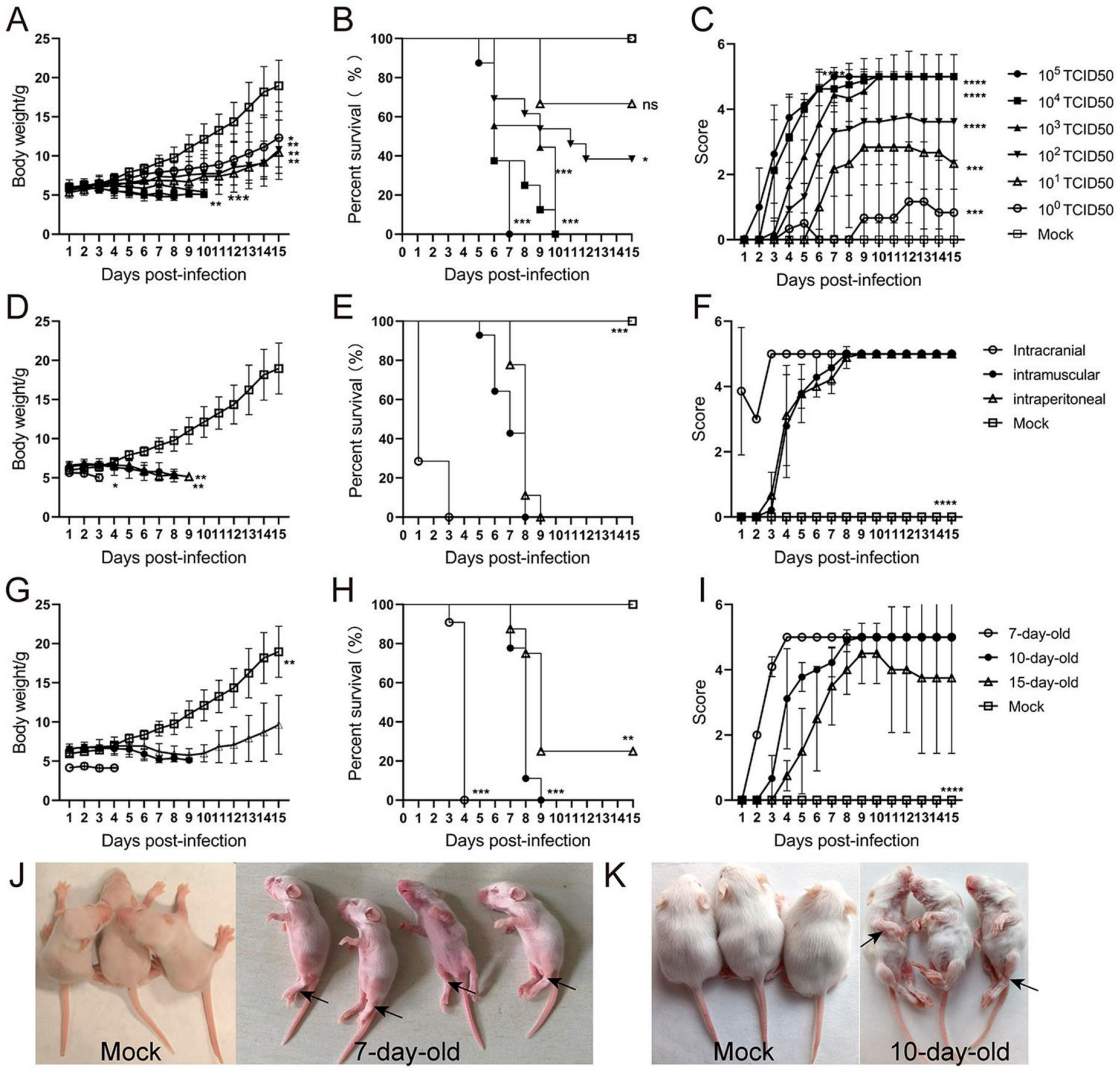
Establishment of the CVA6 infection mouse model. Ten-day-old ICR mice (*n* = 10–15 per group) were i.p. inoculated with different doses of CVA6 (10^0^ TCID_50_–10^5^ TCID_50_), respectively. Control animals were administered muscle homogenate supernatant from normal mice instead of virus. The body weights (A, D, G), survival rates (B, E, H), and clinical scores (C, F, I) in each group of mice were measured. Two representative pictures (J, K) of clinical signs (weight loss, reduced mobility, ataxia, and single or double hind limb paralysis) caused by CVA6 in mice. *P < 0.05; **P < 0.01; ***P < 0.001; ****P < 0.0001; ns, non-significant result.

To determine a suitable infectious dose of CVA6, 10-day-old ICR mice were i.p. inoculated with an undiluted stock of CVA6 (10^5^ TCID_50_) and it’s 10-fold serial dilutions. The results showed that mice infected with doses of 10^3^ ∼ 10^5^ TCID_50_quickly died within 10 dpi. When the infectious dose was reduced to 10^2^ TCID_50_and10^1^ TCID_50_, several mice showed inactive, shortness of breath and neurological symptoms of ataxia, lethargy, and limb paralysis at 11 dpi and 9 dpi, and the ultimate survival rate were 40% and 60%, respectively. In contrast, the mock-infected mice were healthy throughout the experiment (Figure 2(A–C)). These results indicated that there was a significant dose–response effect between the infectious dose of CVA6 and the mortality rate of the mice. Finally, the standard infectious dose of CVA6 was determined as 10^4^ TCID_50_ per mouse. Next, to select a suitable inoculation route, 10-day-old ICR mice were infected via i.p., i.c., or i.m. with10^4^ TCID_50_ of CVA6. As shown in Figure 2(D–F), all mice inoculated via i.m. and i.p. routes became sick at 4 dpi, which resulted in a 100% mortality rate. However, the mice were infected with CVA6 via i.c. route were dead at 1 dpi and resulted in a 100% mortality rate, which differed greatly from the i.p. and i.m. routes. These results indicated that both the i.p. and i.m. routes were suitable for CVA6 infection. For Enterovirus infection pathway, the i.p. route was used as the standard route of infection of CVA6 in the following experiments. To determine the age susceptibility in CVA6 infection, we inoculated 10^4^ TCID_50_ CVA6 to mice at different ages (7, 10, and 15 days) via i.p. route. CVA6 infection resulted in rapid disease onset and a short survival time in 7-day-old mice. Mice younger than 15 days were more susceptible to CVA6 infection, implying age dependency. The average clinical scores of the 10-day-old mice were more than 4 after 5 dpi, and all infected mice died between 5 and 8 dpi (Figure 2(G–I)). Therefore, the 10-day-old ICR mice were selected as animal models in the following experiments. Mock-infected mice inoculated with muscle homogenate supernatant from normal mice were healthy throughout the experiment. Clinical signs of weight loss, reduced mobility, and neurological signs, such as ataxia, lethargy and limb paralysis in CVA6-infected mice are shown in Figure 2(J,K), and these manifestations are similar to human infection.

### CVA6 triggers inflammation-associated CNS damage

**Figure 3.**
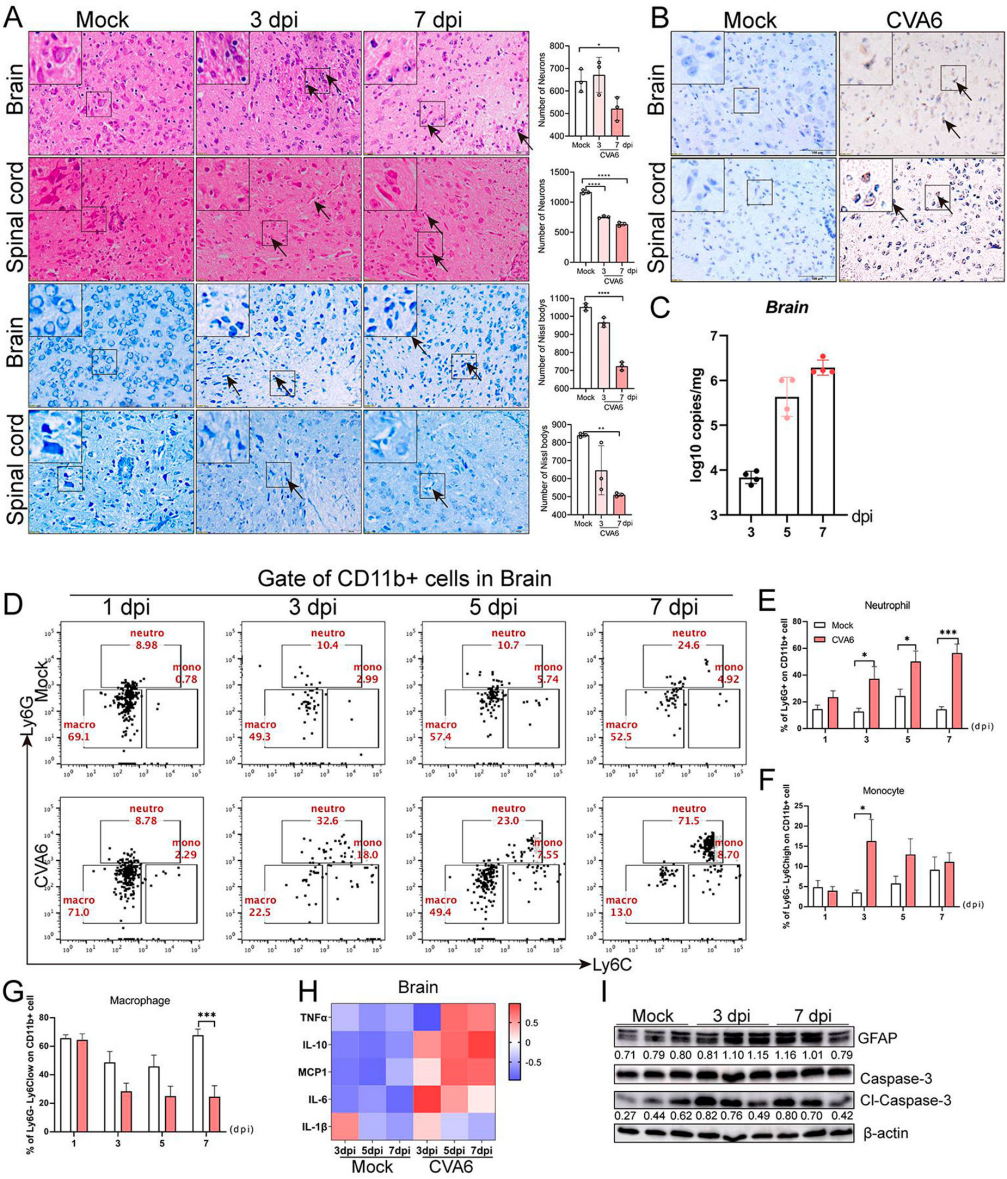
CVA6 triggers inflammation-associated CNS damage. (A) H&E and Nissl’s staining of brain and spinal cord slices. Number of neurons were count at 3 dpi or 7 dpi. (B) The CVA6 antigens in brain and spinal cord tissues were determined by IHC. (C) The copy number of VP1 genome in brains at 3 dpi, 5 dpi and 7 dpi. (D) The number of inflammatory cells in the brains of CVA6 infected and uninfected mice. (E, F, G) Statistical results of neutrophils, monocytes and macrophages in the brains of CVA6 infected and uninfected mice. (H) The expression levels of cytokines (TNFα, IL-10, MCP-1, IL-6, IL-1β) in grinding supernatant of brains measured by ELISA. (I) The relative expression of GFAP and apoptosis proteins (cleaved-Caspase-3) in the brains of CVA6 infected and uninfected mice. *P < 0.05; **P < 0.01; ***P < 0.001; ****P < 0.0001.

To deeply understand the mechanism of neurological manifestations caused by CVA6 infection, we performed the histopathological analysis of the brains and spinal cords and the detection of related indicators. As shown in the Figure 3(A), the number of neurons (Nissl’s bodies) in the brains and spinal cords were significantly decreased at 3 dpi or 7 dpi. Viral antigens were observed in the slices of brains and spinal cords from CVA6-infected mice at 7 dpi (Figure 3(B)**)**, and we also determined viral loads (Figure 3(C)**)** in the brain tissues, indicating the virus invades nervous system. In addition, we found neutrophil and monocyte infiltration in the brain tissues of infected mice. As shown in Figure 3(D–F), the number of neutrophils and monocytes was significantly increased in the brains of CVA6-infected mice at indicated time points, while the number of macrophages was decreased (Figure 3(G)), relative to mock-infected mice. The expression levels of cytokines (e.g. IL-10, MCP-1, IL-6) were also increased in CVA6-infected brains (Figure 3(H)). To further evaluate CNS damage, we detected GFAP and cl-Caspase-3 expression in the brains and found CVA6 infection led to activation of astrocytes and apoptosis of brain cells (Figure 3(I)). These results indicate that severe pathological damage, inflammatory cell infiltration and apoptosis may be the causes of nervous system injury.

### Systemic manifestations triggered by CVA6

**Figure 4.**
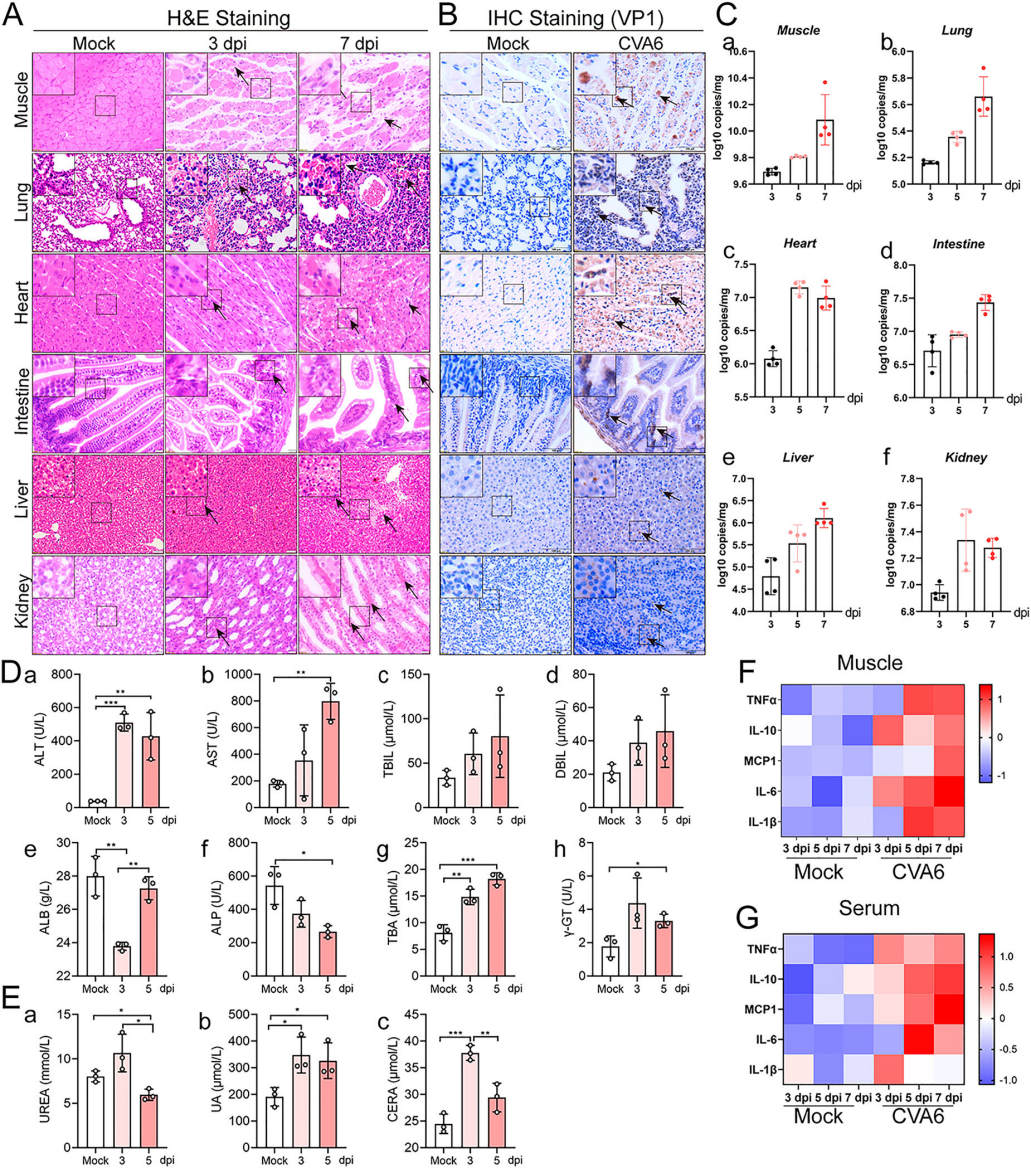
Systemic manifestations triggered by CVA6. (A) The pathology changes of skeletal muscles, lungs, hearts, intestines, livers and kidneys were determined by H&E staining. The black arrows indicate characteristic lesions. (B) The viral antigens were diffusely distributed in all the isolated tissues; no antigen was detected in the control group. The black arrows indicate locations of antigens. (C) The copy number of CVA6 VP1 in muscles (C-a), lungs (C-b), hearts (C-c), intestines (C-d), livers (C-e) and kidneys (C-f) at 3 dpi, 5 dpi and 7 dpi were determined by quantitative PCR. Results are normalized by β-actin from the same organs/tissues. (D) Liver functions (ALT (D-a), AST (D-b), TBIL (D-c), DBIL (D-d), ALB (D-e), ALP (D-f), TBA (D-g) and γ-GT (D-h)) and (E) renal functions (UREA (E-a), UA (E-b), CERA (E-c)) in serums of mice at 3 dpi and 5 dpi were detected by automatic biochemical analyzer. (F) The expression levels of cytokines (TNF-α, IL-10, MCP-1, IL-6, IL-1β) in grinding supernatant of muscles in mice were measured by ELISA. (G) The concentrations of cytokines (TNF-α, IL-10, MCP-1, IL-6, IL-1β) in serums of mock and CVA6 infected mice were measured by ELISA. *P < 0.05; **P < 0.01; ***P < 0.001; ****P < 0.0001.

In order to further evaluate the systemic damage of CVA6 infection in mice, we performed the histopathological analysis of multiple organs and the detection of related indicators. We observed hind limb skeletal muscle fibres exhibited severe necrosis, rupture, and inflammatory cell infiltration (Figure 4(A)). There was inflammatory pneumonia, presenting with massive erythrocytes leakage, which is typical clinical manifestation of pulmonary hemorrhage in human infection (Figure 4(A)**)**. In addition, intestinal necrosis was observed during the late phase of infection (7 dpi), and viral myocarditis characterized with a large number of lymphocytic and mononuclear cell infiltration and myocardial rupture was displayed in CVA6-infected mice (Figure 4(A)**)**. CVA6 VP1 antigens and viral loads were detected in muscle fibre cells of skeletal muscles, the alveolar cells of lungs, cardiomyocytes of heart, intestinal epithelial cells of intestinal derived from CVA6-infected mice (Figure 4(B,C)). CVA6 also triggered hepatitis and liver necrosis (Figure 4(A)), and viral antigens and viral loads were also detected in livers of CVA6-infected mice (Figure 4(B,C-e)). Consistent with the liver damage, liver function was heavily impaired at 5 dpi, as seen by the high serum levels of alanine aminotransferase (ALT), aspartate aminotransferase (AST), total bile acid (TBA), gamma-glutamyl transpeptidase (γ-GT), and the low serum levels of alkaline phosphatase (ALP) (Figure 4(D-a-h)). Moreover, hyperemia and edema (Figure 4(A)**)**, VP1 antigens distribution (Figure 4(B)), and viral loads (Figure 4(C-f)) were detected in kidneys of infected mice. Slight changes in kidney function were observed at 3 or 5 dpi, as seen by the high serum levels of UREA, UA, and CERA (Figure 4(E-a-c)). The concentrations of major cytokines (TNF-α, IL-10, MCP-1, IL-6, IL-1β) were markedly increased at 3 dpi and/or 5, 7 dpi (Figure 4(F,G)). There were no observable pathological changes in the mock-infected mice. Our results suggest that CVA6 lead to multiple organs damage followed by systemic inflammation.

### Oral inoculation of mouse-adapted CVA6 results in mortality

**Figure 5.**
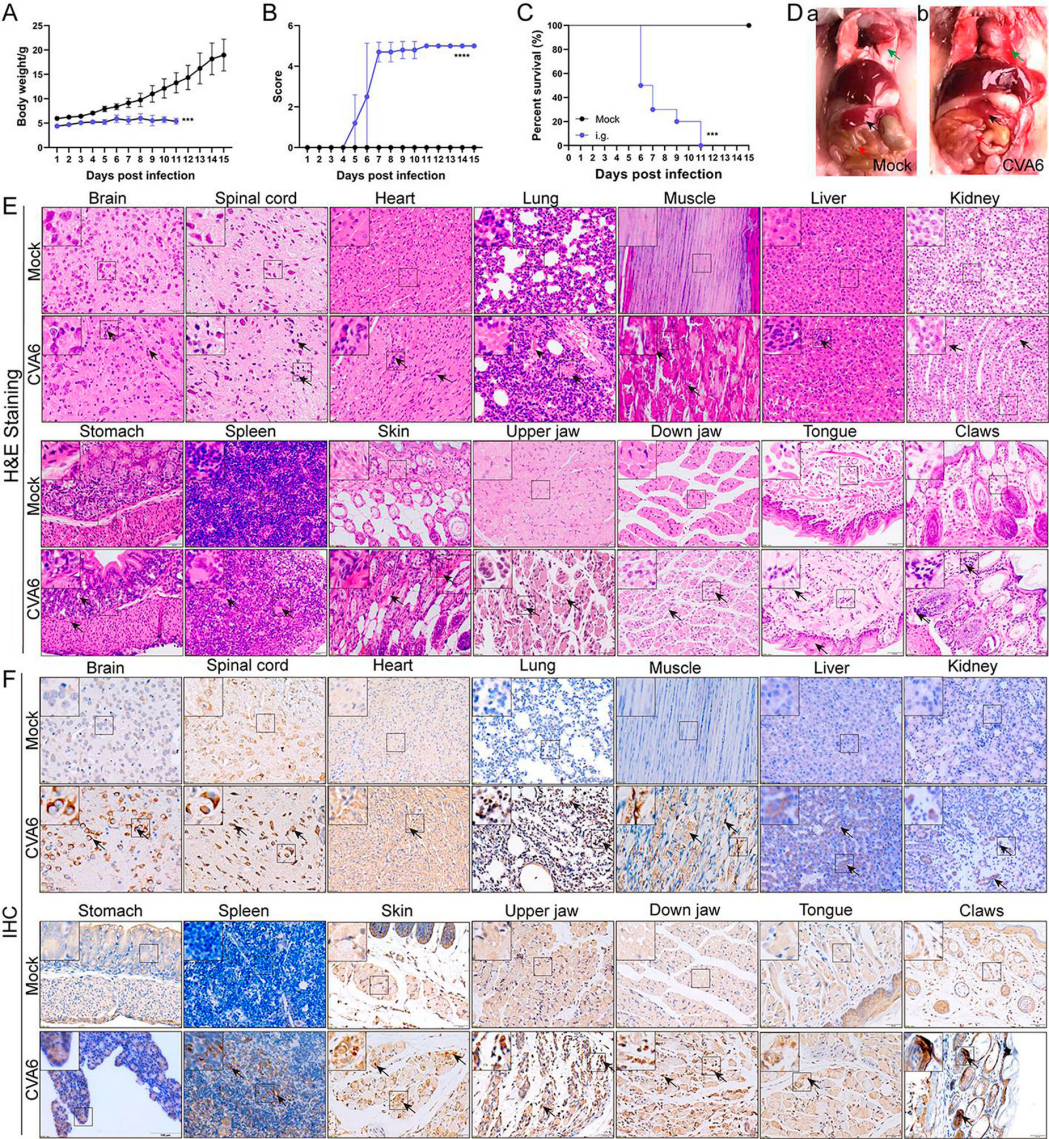
Oral inoculation of mouse-adapted CVA6 results in mortality. Ten-day-old ICR mice (*n* = 10–15 per group) were i.g. inoculated with doses of CVA6 (10^5^ TCID_50_), respectively. Mock animals were administered muscle homogenate supernatant from normal mice instead of virus. The body weights (A), mean clinical scores (B) and survival rates (C) in each group of neonatal mice were recorded. (D) Representative pictures of organs in mock-infected mice (D-a) and CV6-infected mice (D-b). (E) The pathologies of different organs (brains, spinal cords, hearts, lungs, muscles, livers, kidneys, stomachs, spleens, skins, upper jaws, down jaws, tongues, claws) from mice infected with CVA6. No pathological change was observed in the control group. (F) The viral antigens were distributed in fourteen isolated tissues. No antigen was detected in the control group treated with the muscle homogenate supernatant from normal mice. The black arrows indicate characteristic lesions and locations of antigens. *P < 0.05; **P < 0.01; ***P < 0.001; ****P < 0.0001.

CVA6 is an Enterovirus, and to understand its pathogenesis, the disease should be evaluated after oral infection. In order to develop a CVA6 oral infection model, 10-day-old ICR mice were i.g. inoculated with 10^5^ TCID_50_ CVA6. Our results showed that there was a significant change in body weight via i.g. inoculation, and all infected mice became inactive at 5 dpi and died between 6 and 11 dpi (Figure 5(A–C)). There was obvious exudation of the lung (Figure 5(D), green arrow) from CVA6-infected mice compared with the mock-infected mice. Additionally, the size of spleen (Figure 5(D), black arrow) from CVA6-infected mice became smaller, suggesting CVA6 infection may cause damage to immune function. It was hard to observe food as severe edema (Figure 5(D), red arrow) happened in the intestine of infected mice, possibly caused by decreased appetite and limb paralysis of the mice. As revealed by the results of the pathological and immunohistochemical examinations of mice tissues and organs, CVA6 infection triggered multiple tissues and organs damage in 10-day-old mice, which were consistent with i.p. inoculation (Figure 5(E)). Typical pathological alterations were not identified in the tissues of mock-infected mice. As demonstrated by IHC analysis, VP1 antigens were observed in multiple tissues and organs of the suckling mice infected with CVA6 (Figure 5(F)). It is worth noting that virus antigens were detected in skin (myoepithelial cells), up jaw (myoepithelial cells), down jaw (myoepithelial cells) and tongue (striated muscle cells) (Figure 5(F)). We didn’t detect virus antigens in mock-infected mice. The concentrations of cytokines (e.g. IL-10, TNFα, IL-6) were also increased in tissue lysates and serum samples (Figure S3(A–C)).

### CVA6 leads to immune system activation

**Figure 6.**
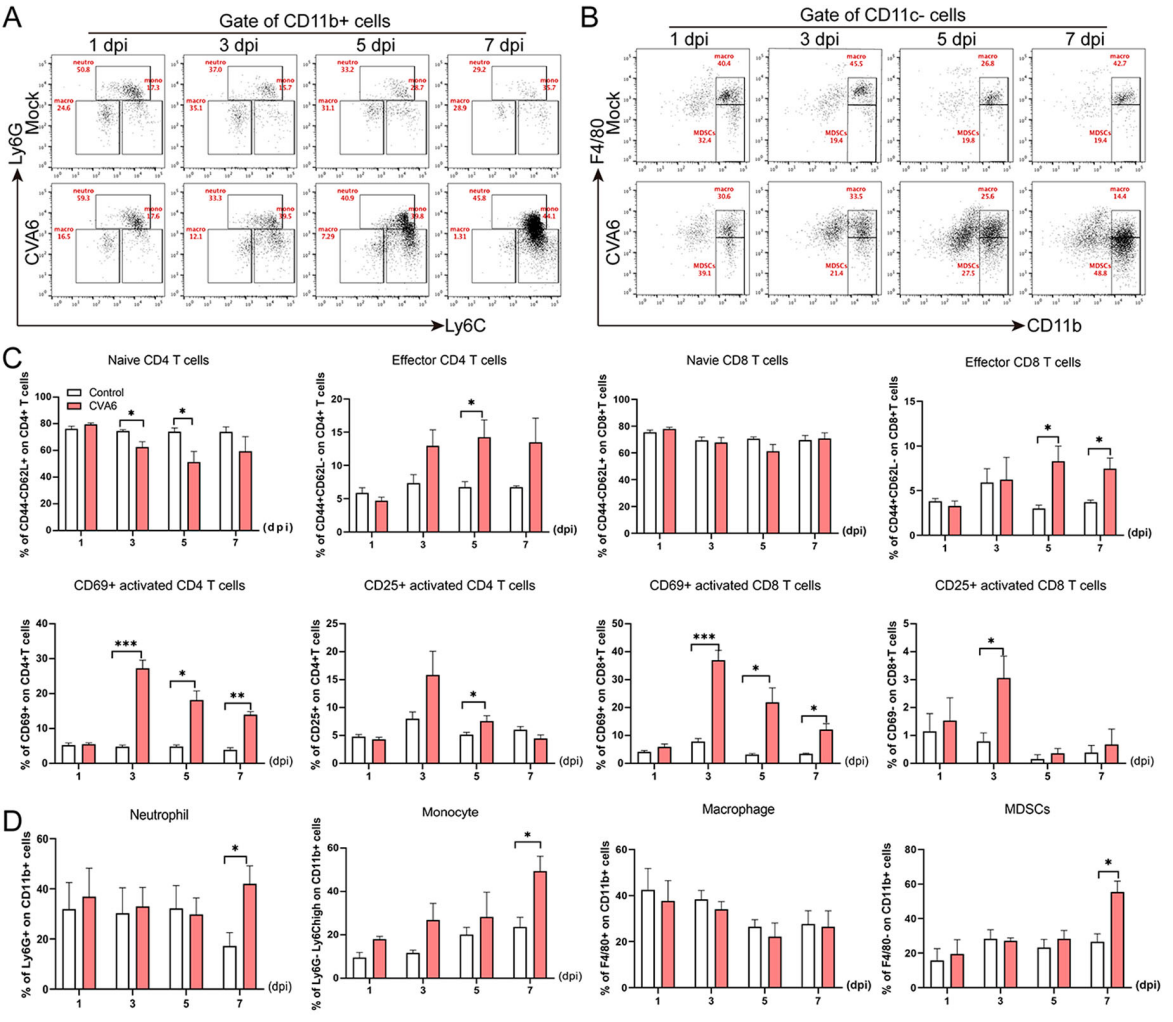
CVA6 leads to immune system activation. The 10-day-old ICR mice were i.p. inoculated with 10^4^ TCID_50_ CVA6 strain. At 1 dpi, 3 dpi, 5 dpi and 7 dpi, control and infected mice (*n* = 7–10) were euthanized. FACS was used to detect the characteristics of immune cells in spleen of CVA6 infected and uninfected mice. (A) The number of neutrophils and monocytes in spleens of mice. (B) The number of macrophages and MDSCs in spleens of mice. (C, D) Statistical results of immune cells and inflammatory cells in the spleens of CVA6 infected and uninfected mice. *P < 0.05; **P < 0.01; ***P < 0.001; ****P < 0.0001.

In order to further investigate the changes of immune function in mice after CVA6 infection, FACS was used to detect the characteristics of immune cells in the spleens of CVA6 infected and uninfected mice. We found depleted B lymphocytes, increased T cells, and the ratios of CD4+ T cells and CD8+ T cells were decreased in mice after CVA6 infection (Figure S1). The results suggest that the immunity of the mice decreases after infection and are not enough to resist the invasion of the virus. However, activated CD4+ T cells and activated CD8+ T cells proliferate in large quantities, which is conducive to recognizing the CVA6 virus. At the same time, effector CD4+ T cells and effector CD8+ T cells proliferated and secreted cytokines to eliminate CVA6 virus (Figure S3(D,E) and Figure 6(C)). We also observed a large number of neutrophils and monocytes in mice infected with CVA6 (Figure 6(A, B, D)). Our results suggest that CVA6 infection triggers immune system activation, which may contribute to systemic inflammation.

### Protective efficacy of ribavirin against CVA6 challenge *in vivo*

**Figure 7.**
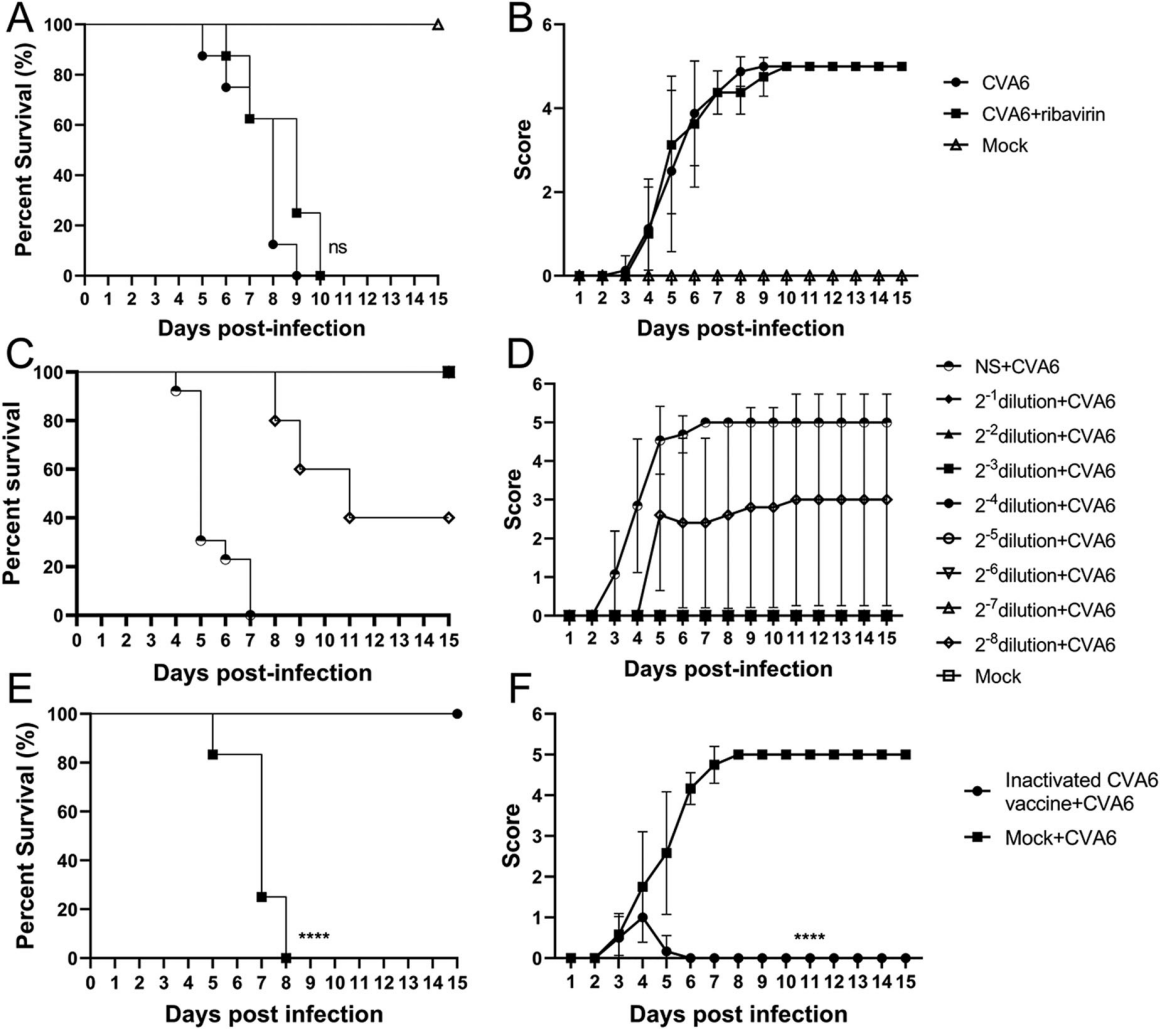
Protective efficacy of ribavirin, CVA6 antiserum and inactivated whole-virus vaccine against CVA6 challenge *in vivo.* (A, B) Effect of ribavirin. (C, D) Effect of passive immunization. (E, F) Effect of active immunization. The survival rates (A, C, E) and Clinical symptoms (B, D, F) were monitored and recorded daily for 15 days after inoculation with CVA6. ****P < 0.0001.

To further characterize the antiviral effects of ribavirin, we employed *in vivo* protection experiments. Ten-day-old mice inoculated orally with lethal doses of CVA6 were injected with therapeutic doses of ribavirin (200 μg/mouse). The clinical symptoms of the mice were under active observation, and the mortality rates were recorded. The mock group that received no treatment showed obvious symptoms and died in a short time. However, there was no statistically significant difference in survival rates (Figure 7(A)) and clinical scores (Figure 7(B)) between the treatment and control groups. The results suggest that ribavirin can’t provide protection against the lethal dose of CVA6 *in vivo*.

### Protective efficacy of virus antiserum against a CVA6 challenge *in vivo*

To evaluate the protective efficacy of the CVA6 antiserum on the mice by passive immunization, we passively transferred 60 μL of the CVA6 antiserum at original and dilutions of 1:2, 1:4, 1:8, 1:16, 1:32, 1:64, 1:128, 1:256 or the negative serum (NS) into 10-day-old mice, followed by i.g. inoculation with 10^5^ TCID_50_ CVA6. As shown in Figure 7(C,D), mice received control CVA6 antiserum started to present clinical signs of limb paralysis at 3 dpi and totally died at 7 dpi. By contrast, all mice administered with original CVA6 antiserum or dilutions of 1:2, 1:4, 1:8, 1:16, 1:32, 1:64, and 1:128 survived, and no neurological symptoms were observed. Our qRT-PCR results showed that mice received CVA6 antiserum exhibited lower viral loads in spinal cord, lung, muscle, heart, liver, spleen and intestine compared with those received negative serum (Figure S4(B)). We also found that CVA6 antigens were significantly reduced or below detection limits in mice after administration with CVA6 antiserum determined by IHC (Figure S4(A)). Our results suggest that the original CVA6 antiserum provided 100% protection (no fatal infection) to neonatal mice.

### Active immunization with inactivated CVA6 whole-virus vaccine protects 10-day-old mice against infection

To investigate whether active immunization could protect neonates, mice were inoculated with inactivated CVA6 whole-virus vaccine 50 μL on day 3 and day 7. After that, 10-day-old immunized mice were exposed to a lethal dose of CVA6 (10^5^ TCID_50_) via i.g. route. As shown in Figure 7(E,F), after CVA6 infection, the survival rates of mice in the active immunization group was 100%, while the mice without vaccination totally died at 8 dpi. The vaccinated mice only presented mild clinical symptoms with 1 or 2 clinical score within 6 days and then stayed normal. By contrast, the unvaccinated mice showed clinical signs at 2 dpi, and all developed into severity or death at 5–8 dpi. These data suggest that the inactivated CVA6 whole-virus vaccine can generate immune protection in neonatal mice. Whether cross-protective effects were elicited by the CVA6 immune response to afford protection to CVA16, EVA71, and other EVs remains to be determined.

## Discussion

With the inoculation of the EVA71 vaccine, the pathogen spectrum of HFMD has gradually changed [5,33,34]. CVA6-related HFMD has been reported worldwide and poses a great threat to children’s health. Most of the CVA6-related HFMD patients presented as high fever and widespread exanthematous rashes [35], and also presented onychomadesis followed by desquamation of palms and soles [36]. We found that CVA6-infected mice exhibited depilation and pathological lesions of the skin in our murine model. Due to experimental conditions, we did not detect the body temperature of the mice. Severe HFMD patients may be accompanied by vomiting, tremor, convulsion, ataxia, limb weakness, lethargy, shortness of breath, dyspnea, or even death [37,38]. Neurological diseases, such as aseptic meningitis and encephalitis were reported in CV-A6-related HFMD patients [35,38]. In this study, CVA6-infected mice showed inactive, shortness of breath, and neurological complications of ataxia, lethargy, and limb paralysis. To summarize, our mouse model mimics most symptoms of human disease. Therefore, establishing animal models with typical clinical symptoms is one of the important steps to understanding the pathogenesis of CVA6 infection and the development of vaccines, therapeutic antibodies, and antiviral drugs. Recently, several animal models [20,27,28,39] have been reported to evaluate vaccines and antiviral drugs and study the pathogenesis. However, those CVA6 animal models differ from our model in several important respects, namely the dose and route of inoculation, the strain of laboratory mice used, and, critically, the virus strain used. For the first time, we established a stable mouse model capable of causing severe illness in 10-day-old mice via oral infection. However, establishing the orally infected model for CVA6 infection is extremely difficult, and the lethal dose required for infection is relatively high. Compared with other injection routes, the oral infection route better simulates the human infection. Secondly, compared with the age of the mice used previously [20], elderly newborn mice can deliver a higher dose of drugs and are easy to use to evaluate antiviral drugs subsequently. Third, 15-day-old newborn mice were also used in our study, which provides a possibility for the establishment of elderly mice models.

EVs infection can produce a high viremia titre that extends the infection from the primary site in the enteric to the CNS, resulting in diverse pathologies [40,41]. Although the incidence of neurological complications caused by CVA6 was 2% in 2013 and 5% in 2017, the poor prognosis raises widespread concern [40]. Therefore, studying CNS manifestations of CVA6 infection is significant for guiding clinical practice. In this study, we observed severe histopathological injury, glial activation, and inflammatory infiltration after CVA6 infection. Increasing evidence suggests that abnormalities cytokines are associated with CNS complications caused by EVA71 and CVA16 infection [42,43]. After EVA71 infection, susceptible cells and nonspecific immune cells are stimulated first to produce cytokines such as TNF-α and IL-6. These cytokines play an important role in the early control of viral replication and infection. Furthermore, the activation of these cells by cytokines leads to the secretion of inflammatory mediators and cytokines, interferes with viral replication, and kills virus-infected host cells [44]. Our results showed that cytokines were produced and inflammatory cell infiltration (neutrophil and monocyte) in mice brains infected with CVA6. We speculate that the integrity of the blood–brain barrier (BBB) may be damaged permitting cytokines action within the CNS [42]. CNS lesion is the primary inducement of severe clinical symptoms such as poor mental state, lethargy, easy to panic, ataxia, muscle weakness or acute tardy paralysis, frequent convulsions, and other nerve injuries [1]. Dissection of EVA71 infection-related fatal cases revealed that the EVA71 antigens could be detected in neurons of brain stem and spinal cord [45]. Our Data from this study showed that CVA6 replication and CVA6 VP1 antigens could be detected in the brain and spinal cord tissues of mice after CVA6 infection. Therefore, the spinal cord may be a “bridge” for CVA6 virus to invade brain tissue, which is consistent with a previous study of other Enteroviruses [46]. In conclusion, the animal model of CVA6 infection established via i.p. or i.g. inoculation in this study presented neuropathological features and symptoms of nerve injury similar to those of clinical cases.

It is noteworthy that CVA6 spreads to multiple organs and triggers a systemic and lethal disease during CNS damage. 10-day-old ICR mice were highly sensitive to CVA6 and exhibited symptoms similar to severe HFMD cases, such as depilation, lethargy, emaciation, quadriplegia, ataxia, and tachypnea. Our results suggest that CVA6 exhibits a strong muscle tropism, which may be one of the direct causes of paralysis and inability to obtain breast milk in newborn mice, thus accelerating their death. Previous studies confirmed that skeletal muscle was the most important site for CVA6 virus replication [20,28,39,47]. Other studies have suggested that skeletal muscle is the leading site for the replication of Enterovirus infection, providing the source of virus for CNS infection [48,49]. In this study, lung hyperemia, red blood cell leakage of capillary, and lung interstitial red blood cell increase were observed in both i.p. and i.g. infection suckling mice, which is similar to the results of other animal models and the investigation of respiratory disease in clinical cases [38]. This suggests that the pulmonary lesions induced by CVA6 infection are independent of the infection route. While pulmonary edema has generally been believed to be neurogenic, the possibility that pulmonary failure may be caused in part by myositis involved in lung respiration [50]. In addition, we have reported that CVA2 led to heart injury in a neonatal mouse model, which might be related to viral replication, increased expression levels of MMP-related enzymes and excessive inflammatory responses [30]. Likewise, we observed heart injury in CVA6-infected mice. A previous study reported that the prognosis of EVA71 infected critical infection was worse once acute kidney injury (AKI) developed [51]. We here found severe renal hyperemia and increased UREA, UA, and CERA in CVA6 mice. In addition, urinary retention was observed in CVA6-infected mice. Liver damage caused by Enterovirus infection is rarely reported. We first observed viral hepatitis in CVA6-infected mice. Serum ALT and AST were both increased in CVA6-infected mice. At 7 dpi, serum ALT decreased while TBIL and DBIL increased progressively, indicating “biliary enzyme separation,” which might be a precursor of liver necrosis. Finally, the concentrations of major cytokines (TNF-α, IL-10, MCP-1, IL-6, IL-1β) were markedly increased at 3 dpi and/or 5, 7 dpi. These results suggest that CVA6 causes damage to multiple organs followed by systemic inflammation.

Mouse models for CVA6 infection have been reported, but those models cannot be consistently orally infected. Those models either inject the viruses through i.p. and i.m. routes [20], or neonatal animals (1-day-old, 3-day-old, 5-day-old) [28,52] that are generally too difficult to handle. In comparison, our model has several advantages: (i) It uses the oral route, which mimics the natural route of infection; (ii) 10-day-old ICR mice are old enough for oral injection without causing physical injury to the animal; (iii) The older mice are tolerable to a larger dose of the antiviral drug. With a sufficiently high dose of CVA6 (10^5^ TCID_50_), the ICR mice invariably developed squamous lesions in the paw, skin and oral cavity, pulmonary edema and encephalomyelitis that strikingly resembled complicated HFMD. Intriguingly, the involvement of oral cavity and paws corresponded so well with the mucocutaneous lesions in HFMD. Therefore, the model we established can be used to better understand virus entry and spread, pathogenesis, and host immune responses.

Studies have shown that cellular immune dysfunction occurs in children with HFMD [53]. When infants were infected with EVA71, T cell subsets changes in peripheral blood might be correlated with the disease severity to a certain extent [54]. However, there have not been reported cellular immune changes in HFMD patients with CVA6 infection. In our study, FACS was used to analyze the changes of T cells in peripheral immune organs of CVA6-infected mice. Our results showed T cell activation and the increased number of neutrophils, monocytes, and MDSCs in the spleens of CVA6-infected mice. Our results suggest CVA6 triggers systemic immune activation. We also found that a large number of B cells are depleted by the virus (Figure S1), suggesting the proliferation and maturation of B cells were inhibited after CVA6 infection.

Thus far, there is no effective treatment strategy for HFMD and only i.v. immunoglobulin has been licensed as an anti-EVA71 therapy; however, its efficacy is undemonstrated [55,56]. In the present study, ribavirin therapy fails to relieve the disease severity. However, another study found that ribavirin suppressed viral replication to extremely low levels *in vitro* (<5%) with a 60% survival rate *in vivo* and significantly shortened the duration of the disease [28]. We next study whether antiviral serum could be used to control CVA6 infection. We found the CVA6 antiserum showed 100% protection against a lethal viral challenge, indicating that neutralizing antibodies may play an essential role for *in vivo* protection. Zhang et al. reported that no protective effects or diminution of disease severity could be provided to neonatal mice with paralysis of the one or both hind limbs [28]. However, more CVA6 clinical isolates and genotypes need to be examined to determine the extent of neutralization by the CVA6 antiserum. And further studies are needed to study whether viral antiserum can reverse disease severity. Immunization is considered to be the most effective tool with which to control HFMD epidemics. However, experimental EVA71 and CVA16 vaccines could not induce heterologous cross-protection against infection with CVA6 [57]. It is important for the development of a CVA6 vaccine to verify that anti-CVA6 serum has protective functions *in vivo* similar to those of EVA71 and CVA16 antisera. In the present study, we successfully evaluated the protective efficacy of inactivated CVA6 candidate vaccine based on the clinical symptoms and survival rates of infected neonatal mice. Indeed, our results indicate that the candidate inactivated CVA6 vaccines can protect mice from CVA6 infection. Moreover, the protective efficacy of inactivated CVA6 candidate vaccine has been successfully evaluated by immunizing newborn mice [47]. In the future, an appropriate immunization schedule will be administered on adult female mice to determine if candidate vaccines can induce sufficiently high levels of expression of protective neutralizing antibodies and whether immunized adult female mice can transfer protective neutralizing antibodies to their progeny.

In conclusion, we have demonstrated that the mouse adapted CVA6 strain exhibits continuous virulence. This unique mouse model can further our understanding of neurotropism, neurovirulence, as well as systemic manifestations of CVA6 infection. Moreover, our novel CVA6 murine model provides a valuable tool for future research on vaccines and antiviral agents against CVA6.

## Supplementary Material

Supplemental Material

## Data and materials availability

All data generated in this study are available from the corresponding author upon reasonable request. The live CVA6 used to establish murine model (GenBank Accession number: OM179765) was isolated from stool sample of a HFMD case.
