# Supplementary figures and images for: A mouse-adapted CVA6 strain exhibits neurotropism and triggers systemic manifestations in a novel murine model
Source: Emerg Microbes Infect. 2022 Sep 26;11(1):2248–63. doi: 10.1080/22221751.2022.2119166 (PMC9518251; doi:10.1080/22221751.2022.2119166)

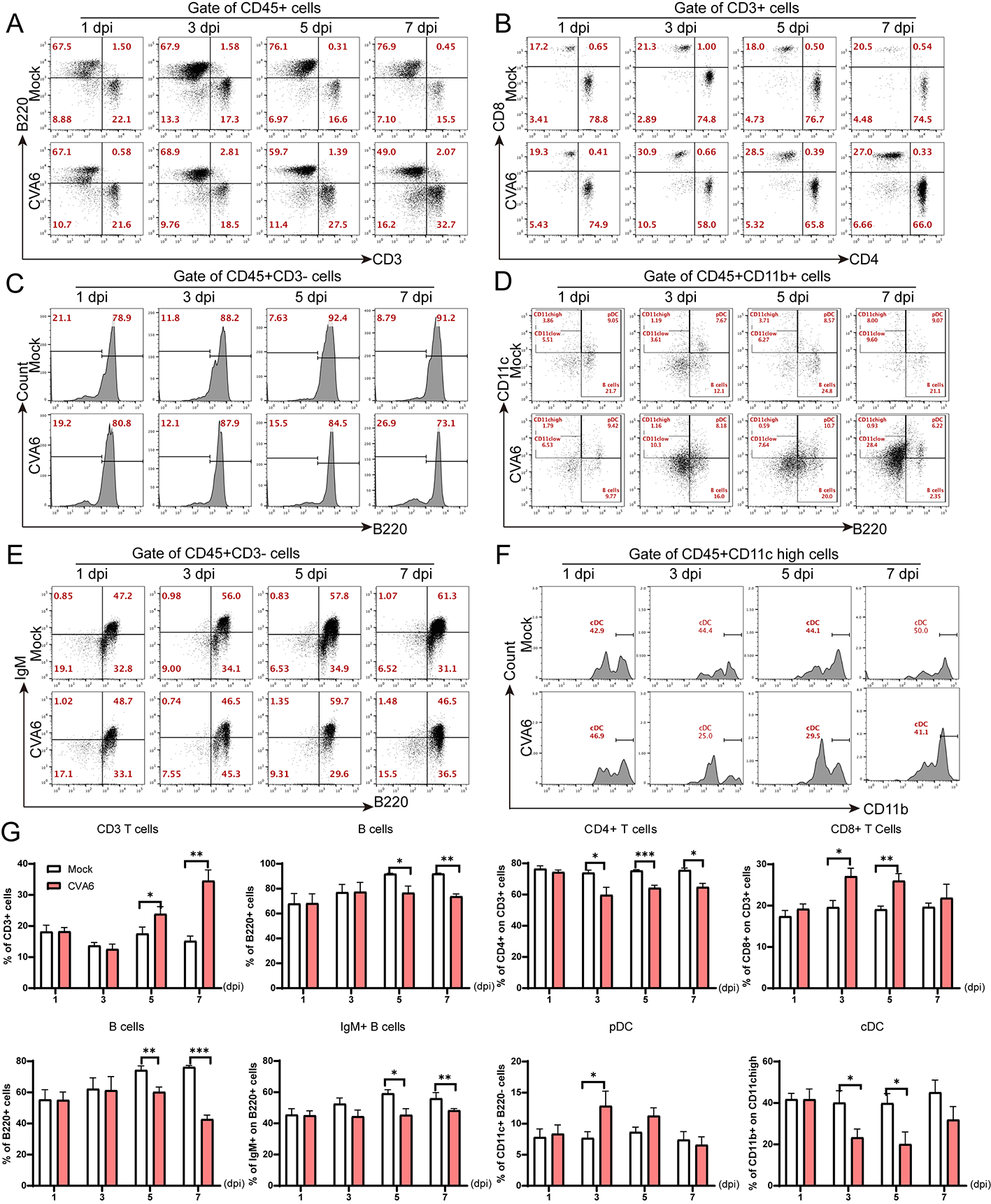

Supplement: Supplemental Material [file TEMI_A_2119166_SM1306.zip › Figure_S1.tiff]

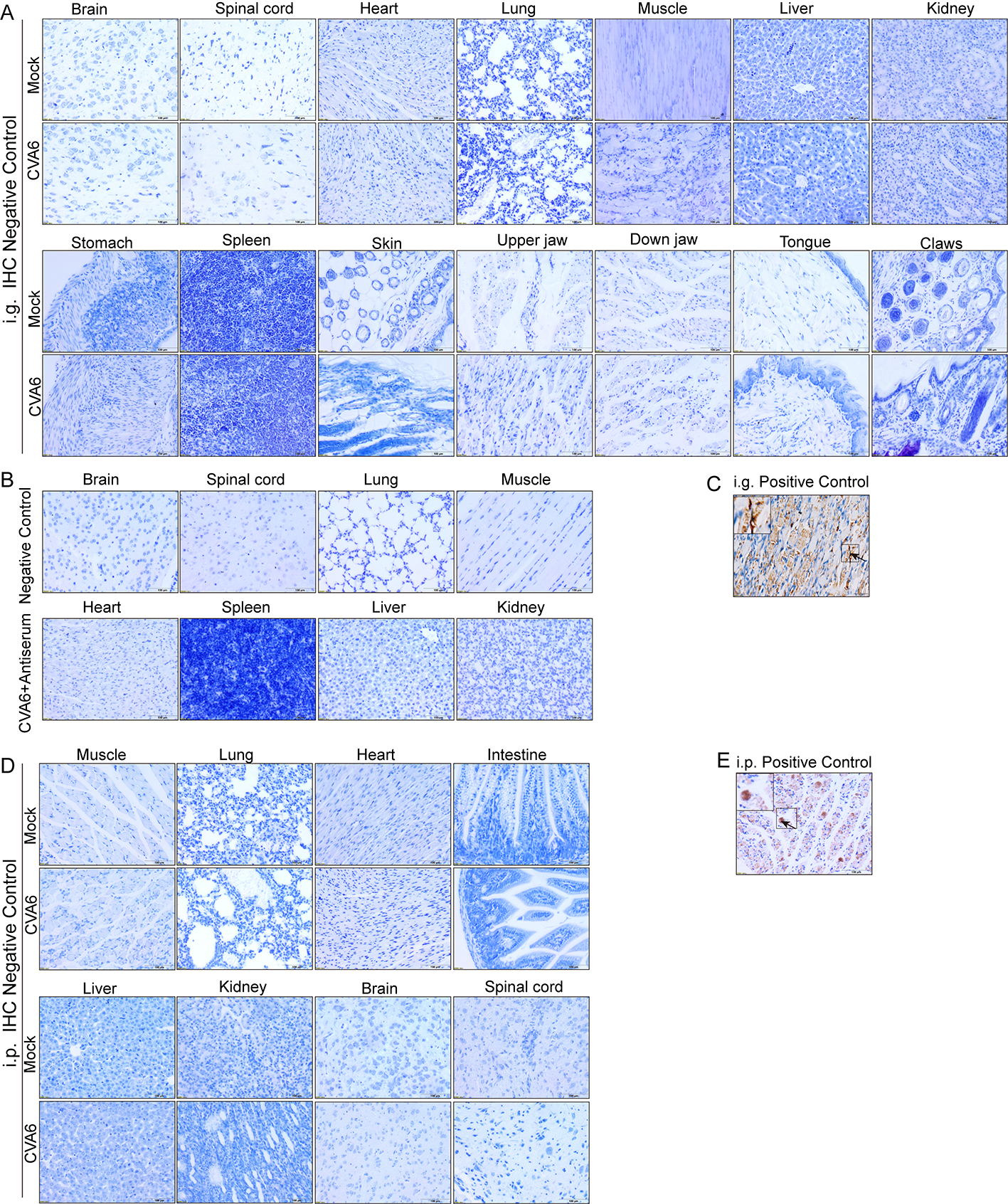

Supplement: Supplemental Material [file TEMI_A_2119166_SM1306.zip › Figure_S2.tiff]

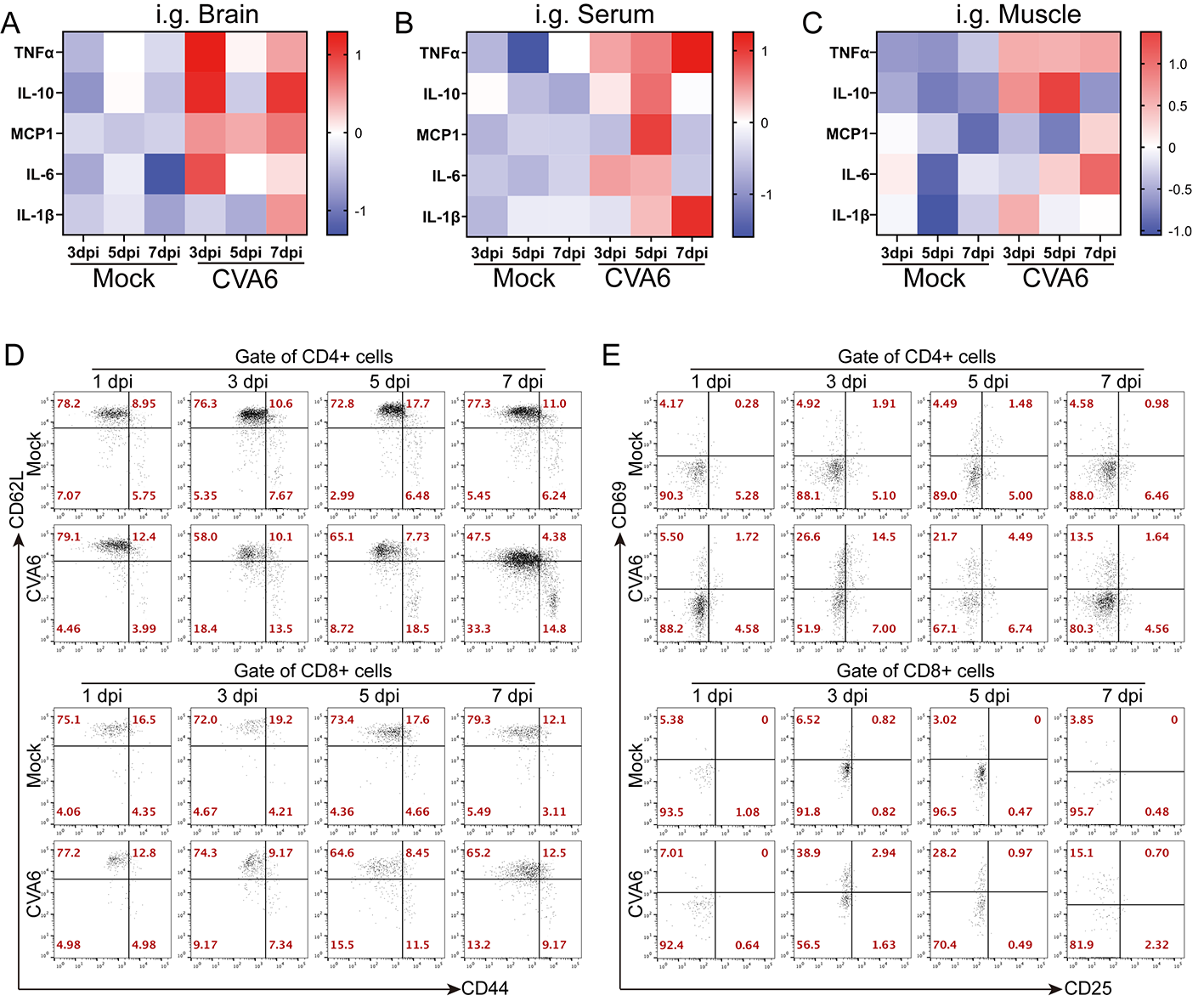

Supplement: Supplemental Material [file TEMI_A_2119166_SM1306.zip › Figure_S3.tiff]

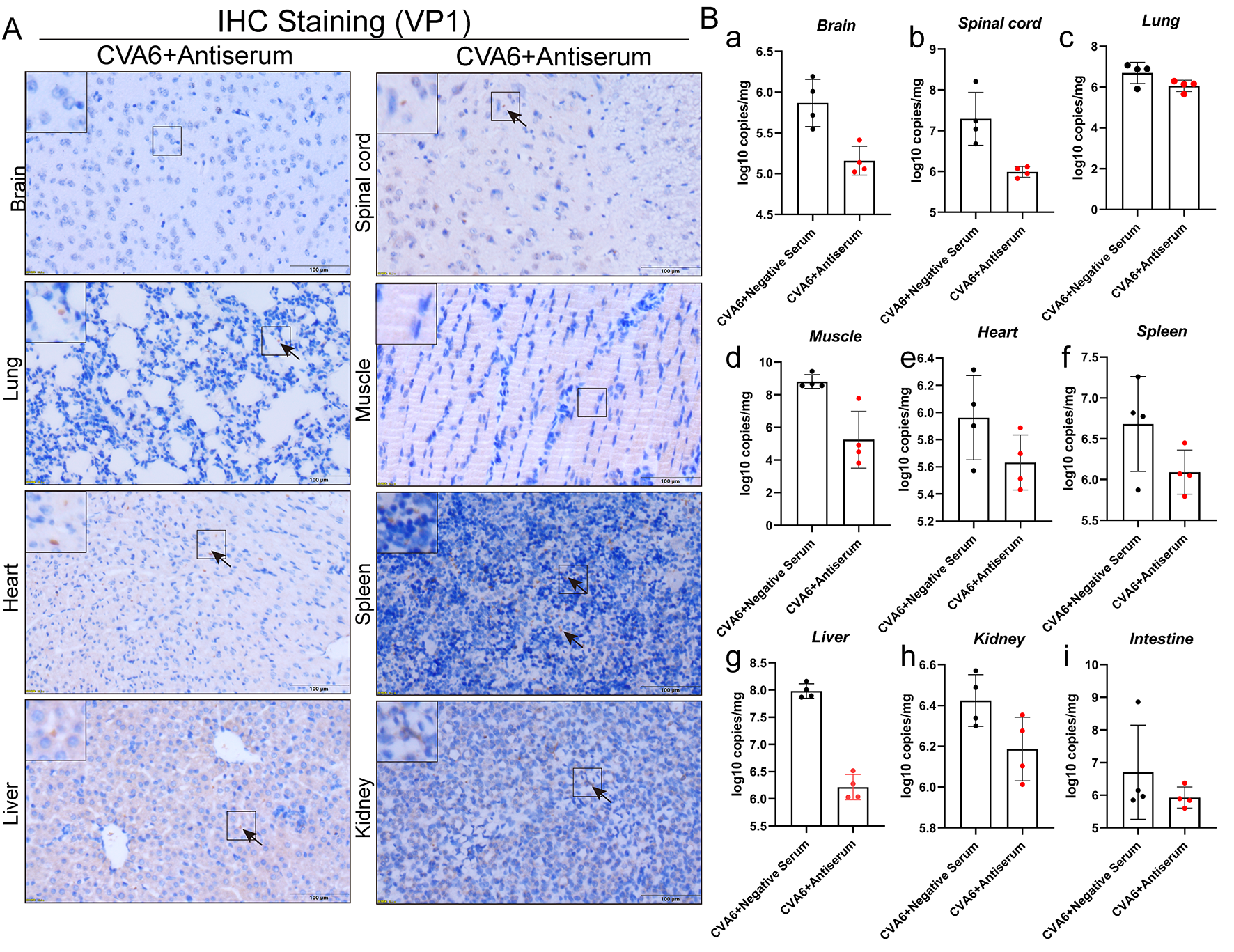

Supplement: Supplemental Material [file TEMI_A_2119166_SM1306.zip › Figure_S4.tiff]
